# Genomic Analysis Based on Chromosome-Level Genome Assembly Reveals an Expansion of Terpene Biosynthesis of *Azadirachta indica*

**DOI:** 10.3389/fpls.2022.853861

**Published:** 2022-04-18

**Authors:** Yuhui Du, Wei Song, Zhiqiu Yin, Shengbo Wu, Jiaheng Liu, Ning Wang, Hua Jin, Jianjun Qiao, Yi-Xin Huo

**Affiliations:** ^1^Key Laboratory of Molecular Medicine and Biotherapy, School of Life Sciences, Beijing Institute of Technology, Beijing, China; ^2^National Engineering Laboratory for Efficient Utilization of Soil and Fertilizer Resources, College of Resources and Environment, Shandong Agricultural University, Tai’an, China; ^3^Key Laboratory of Systems Bioengineering (Ministry of Education), School of Chemical Engineering and Technology, Tianjin University, Tianjin, China; ^4^SynBio Research Platform, Collaborative Innovation Centre of Chemical Science and Engineering (Tianjin), Tianjin University, Tianjin, China; ^5^Tobacco Research Institute, Chinese Academy of Agricultural Sciences, Qingdao, China

**Keywords:** *Azadirachta indica*, chromosome-level assembly, comparative genomics, terpene biosynthesis, genome evolution

## Abstract

*Azadirachta indica* (neem), an evergreen tree of the Meliaceae family, is a source of the potent biopesticide azadirachtin. The lack of a chromosome-level assembly impedes an in-depth understanding of its genome architecture and the comparative genomic analysis of *A. indica*. Here, a high-quality genome assembly of *A. indica* was constructed using a combination of data from Illumina, PacBio, and Hi-C technology, which is the first chromosome-scale genome assembly of *A. indica*. Based on the length of our assembly, the genome size of *A. indica* is estimated to be 281 Mb anchored to 14 chromosomes (contig N50 = 6 Mb and scaffold N50 = 19 Mb). The genome assembly contained 115 Mb repetitive elements and 25,767 protein-coding genes. Evolutional analysis revealed that *A. indica* didn’t experience any whole-genome duplication (WGD) event after the core eudicot γ event, but some genes and genome segment might likely experienced recent duplications. The secondary metabolite clusters, TPS genes, and CYP genes were also identified. Comparative genomic analysis revealed that most of the *A. indica*-specific TPS genes and CYP genes were located on the terpene-related clusters on chromosome 13. It is suggested that chromosome 13 may play an important role in the specific terpene biosynthesis of *A. indica*. The gene duplication events may be responsible for the terpene biosynthesis expansion in *A. indica*. The genomic dataset and genomic analysis created for *A. indica* will shed light on terpene biosynthesis in *A. indica* and facilitate comparative genomic research of the family Meliaceae.

## Introduction

*Azadirachta indica* (neem) is a member of Meliaceae family, which is extensively studied for its bioactive products (Schmutterer, 1995). It grows natively on the Indian subcontinent and also in other countries such as Egypt and the Kingdom of Saudi Arabia. *A. indica* is a source of abundant limonoids and simple terpenoids which are responsible for its biological activity (Dai et al., 2001). Azadirachtin, the most important active compound in the neem tree, has been intensively studied because of its wide range of insecticidal properties and low toxicity in mammals (Ley, 1994). Additionally, the neem tree extracts also exhibit many pharmaceutical functions, such as anti-inflammatory, anticancer, antimicrobial, and antidiabetic activities (Soares et al., 2014; Abdelhady et al., 2015). A lot of studies have focused on the synthesis of azadirachtin, including chemosynthesis, hairy root culture, cell line culture, and callus culture (Veitch et al., 2007; Srivastava and Srivastava, 2013; Mithilesh and Rakhi, 2014; Rodrigues et al., 2014). However, these methods are either of low extraction efficiency or not environmentally friendly. Therefore, the reconstruction of biosynthetic pathway of azadirachtin for heterologous production is an alternative method.

An omics strategy is an effective method to study the biosynthesis of secondary metabolites. Transcriptomes of *A. indica* tissues (stem, leaf, flower, root, and fruit) have been sequenced, which paved the way to the potential synthetic pathway of azadirachtin and gene expression profiles in various organs. Draft genomes have also been sequenced, which led to a basic understanding of the genetic characteristics of *A. indica* (Krishnan et al., 2012, 2016; Kuravadi et al., 2015). However, the lack of a chromosome-level genome sequence has hindered a full understanding of the secondary metabolite biosynthesis and the evolution of *A. indica*. In addition, Meliaceae are known to produce around 1,500 structurally diverse limonoids, which have agricultural and medical values (Hodgson et al., 2019). A chromosome-level genome is essential for genome-wide studies of the Meliaceae family.

In this study, the first chromosome-level genome of *A. indica* was assembled through a combination of Illumina, PacBio, and Hi-C technology. Based on the assembled genome sequence and annotation, we characterized the history of gene and whole-genome duplication (WGD) events, as well as the evolution of secondary metabolite clusters and resistance genes. These results improved the understanding of the genomic architecture of *A. indica*. This chromosome-level genome assembly can be used as a new reference genome for *A. indica*, laying a substantial foundation for further genomic studies.

## Materials and Methods

### Plant Material, DNA Preparation, and Genome Sequencing

Fresh tissues of *A. indica* were randomly collected from a locally grown tree in the Liufang Yuan park of Hainan University (100.61438 E, 36.28672 N), Hainan Province, China. Fresh leaves were collected to isolate genomic DNA of *A. indica* for *de novo* sequencing and assembly. Genomic DNA was extracted from leaves of *A. indica* using the DNAsecure Plant Kit (TIANGEN, Biotech Co., Ltd., Beijing, China). For Illumina sequencing, a paired-end library with an insert size of 270 bp was generated and sequenced on the Illumina HiSeq X Ten platform. For PacBio sequencing, a 20 kb insert library was generated and sequenced on the PacBio RSII platform.

### Genome Assembly

First, Canu v2.0 (Koren et al., 2017) software was used to correct and assemble raw PacBio sequencing reads, and 886 contigs with N50 ∼ 6 M were assembled by Canu. In addition, we performed a round of polishing on the assembled contigs using the RACON (Vaser et al., 2017) with the PacBio long reads, and the polished contigs were further corrected two rounds on the genome-wide base-level by Pilon v1.21 (Walker et al., 2014) with the Illumina short reads. 870 contigs were left after error correction with RACON and Pilon software. Genome size of *A. indica* was estimated by flow cytometry (Pellicer and Leitch, 2020).

### Chromosome Assembly Using Hi-C

For Hi-C sequencing, a 150 bp paired-end library was generated and sequenced on the Illumina HiSeq X Ten platform. Bowtie2 (Langmead and Salzberg, 2012) with the default parameters was used to map the clean reads to the *A. indica*. HiC-Pro v2.11.1 (Servant et al., 2015) was used to map the Hi-C sequencing reads to the assembled draft genome and detect the valid contacts. Then we used ALLHIC v0.9.12 (Zhang et al., 2019) to cluster contigs into chromosome-scale scaffolds based on the relationships among valid contacts.

### Assessment of Genomic Integrity

The draft genome sequence of *A. indica* (GCA_000439995.3) was downloaded from NCBI as a reference. The accuracy and integrity of the genome assembly was evaluated using BUSCO v3.0.2, based on the OrthoDB^1^ database. LTR Assembly Index (LAI) scores were calculated by LTR_Retriever (v2.8) with the default parameters (Ou and Jiang, 2018; Ou et al., 2018). The transcriptomic NGS short reads from 5 tissues of *A.indica* (SRR12709585, SRR12709584, SRR12709583, SRR12709582, and SRR12709581) (Wang et al., 2020) were mapped against the assemblies using Hisat2 (Kim et al., 2015) with default parameters. The genomic NGS short reads were also mapped to the assemblies using Bowtie2. Finally, the collinearity analysis between our assembly and GCA_000439995.3 was performed with Minimap2^2^ and dotPlotly.^3^

### Repetitive Elements

We identified repetitive elements through both RepeatModeler v1.0.8 (Price et al., 2005) and RepeatMasker v4.0.7 (Tarailo-Graovac and Chen, 2009). The LTRs of *A. indica* were identified by using LTRharvest v1.6.1 (Ellinghaus et al., 2008) and LTR_Finder v1.05 (Xu and Wang, 2007). LTR_retriever v2.8.7 (Ou and Jiang, 2018) was used to integrate the results of LTRharvest and LTR_Finder. RepeatModeler employed RECON v1.08 and RepeatScout v1.0.5 to predict interspersed repeats and then combined the repeat sequences from LTR-retriever with the repeat sequences from RepeatModeler to be the local repeat library. To recover the repeats in the *A. indica* genome, a homology-based repeat search was conducted by using RepeatMasker with the *ab initio* repeat database and Repbase.^4^

### Non-coding RNAs

Non-coding RNAs were detected through searching against various RNA libraries. Reliable tRNA positions were searched *via* tRNAscan-SE v1.3.1 (Lowe and Eddy, 1997). Small nuclear RNAs (snRNAs) and microRNAs (miRNAs) were searched by using INFERNAL v1.1 (Nawrocki and Eddy, 2013) against the Rfam (Griffiths-Jones et al., 2005) database.

### Gene Prediction

Homology annotation was performed using genomes of three representative species, including *Citrus sinensis* (Xu et al., 2013), *Theobroma cacao* (Argout et al., 2011), and *Acer yangbiense* (Yang et al., 2019). The TBLASTN software (Camacho et al., 2009) was used to align the protein sequences of these species to *A. indica* genome sequence, with an *E*-value ≤ 1e-5. The exact gene structures were predicted using GeneWise 2.2.0 (Birney et al., 2004) according to the TBLASTN results. We used Cufflinks v2.2.1 (Trapnell et al., 2012) to preliminarily identify gene structures based on the RNA-seq data. *ab initio* annotation was performed using Augustus v3.2.2 (Stanke et al., 2004) and SNAP (Korf, 2004) with the repeat-masked genome sequences. All genes predicted from the three annotation procedures were integrated with MAKER (Holt and Yandell, 2011) software.

### Functional Annotation

The protein sequences of the consensus gene set were aligned to four protein databases, including NR,^5^ InterPro,^6^ Swiss-Prot,^7^ and EggNOG (Powell et al., 2012), for predicted gene annotation. The physically clustered specialized metabolic pathway genes were identified by the PlantiSMASH analytical pipeline (Kautsar et al., 2017). Plant disease resistance (R) genes were predicted by the Disease Resistance Analysis and Gene Ontology (DRAGO) pipeline (Osuna-Cruz et al., 2018).

### Phylogenetic Analysis and Expansion/Contraction of Gene Families

The genome of *A. indica* and 13 other plants were selected for phylogenetic analysis. All-vs.-all BLASTP (Altschul et al., 1997) search results with an *E*-value ≤ 1e-5 were grouped into orthologous and paralogous clusters using OrthoFinder v2.3.7 (Emms and Kelly, 2019). Multiple sequence alignments of all single-copy orthologous gene families were performed by using MUSCLE (Edgar, 2004). The set of single nucleotide polymorphisms (SNPs) presented in each single-copy orthologous gene family was extracted and then integrated according to the arrangement of the genes on the *A. indica* genome. A maximum likelihood (ML) tree was constructed using the integrated SNPs by PhyML v3.1 (Guindon et al., 2009). Divergence time between species was estimated using MCMCtree, which was incorporated in the PAML v4.8 package (Yang, 1997). CAFÉ v3.1 (De Bie et al., 2006) was used to measure the expansion/contraction of orthologous gene families.

### Genome Duplication Analysis

MCScan v0.8 (Tang et al., 2008) package with default parameters was used for the detection of syntenic blocks, defined as regions with more than 5 collinear genes. We aligned the amino acid sequences of syntenic block gene pairs and reciprocal best hits (RBH) gene pairs using MAFFT and further aligned their nucleotide sequences using ParaAT (Zhang et al., 2012). The synonymous substitution rate (*Ks*) values of these gene pairs were calculated using YN model in KaKs_Calculator v2.0 (Wang et al., 2010). The value of *Ks* peak was determinated by the abscissa value of the highest point of the *A. indica Ks* plot. The WGD events of each species were estimated based on the *Ks* distributions. The gene pairs with the median *Ks* < 0.05 were defined as the retained genes from the recent segmental duplication. According to the formula *T* = *Ks*/2*r*, the *Ks* values were converted to divergence times, where *T* is divergence time and *r* is the neutral substitution rate (*r* = 3.39 × 10^–9^). The paralog analysis in *A. indica* genome were performed using RBH from all-vs.-all BLASTp searches using *A. indica* protein sequences. RBHs are defined as reciprocal best BLASTp matches with *e*-value threshold of 1e-5, c-score threshold of 0.3 (Guo et al., 2018).

### Identification and Phylogenetic Analysis of Terpene Synthase and Cytochrome P450 Family Members

Genomes were aligned using HMMER 3.0 search with an *E*-value 1e-5 against the Pfam-A database (02-May-2020) locally. PF01397 (Terpene synthase, N-terminal domain) and PF03936 (Terpene synthase family, metal binding domain) domains were used to identify the members of the TPS gene family. The collection used for phylogenetic analysis consisted of 403 putative TPSs from *A. indica* and other 13 plants and six reported TPSs belonged to TPS- a (AAX16121.1), b (AAQ16588.1), c (AAD04292.1), e (Q39548.1), f (Q93YV0.1), and g (ADD81294.1) subfamilies (Kumar et al., 2018b; Zhou et al., 2020). PF00067 (Cytochrome P450) was used to identify the members of the CYP gene family. Putative CYPs were screened by amino acid length (450 < length < 600) to perform phylogenetic analysis. Protein sequences were aligned using ClustalX in MEGAX using default sets (Kumar et al., 2018a). The ML trees were constructed based on the alignment of TPS and CYP protein sequences using MEGAX software with 100 bootstrap replicates, respectively. The identification of *A. indica*-specific TPS and CYP genes was based on the phylogenetic analysis using other 13 plant genome as the outgroup and a cutoff of 55% identity, which indicated separate subfamily assignment (Liu et al., 2018; Tu et al., 2020).

## Results

### Genome Sequencing and Assembly

To obtain a chromosome-level assembly of *A. indica*, the genome was sequenced using a combination of Illumina, PacBio, and Hi-C methods, and assembled by a hierarchical approach. A total of 110 Gb (providing 188 × genome coverage) Illumina paired-end short reads were produced and the heterozygosity ratio was estimated to be 0.896%. Based on the 21-mer depth distribution of the Illumina short reads, the genome size was estimated to be 165 Mb (Supplementary Figure 1).

We also generated 126 Gb of raw PacBio sequencing reads from the single-molecule real-time (SMRT) sequencing platform, reaching 256 × coverage of the *A. indica* genome (Supplementary Figure 2 and Supplementary Table 1). The total size of the reads assembled from the post-correction genome was 281,629,231 bp with a GC content of 32.2%, consisting of 870 contigs. The contig N50 was 6,039,544 bp, and the longest contig was 15,111,501 bp. Our genome assembly constitutes ∼73.2% of the 385 Mb genome estimated by flow cytometry (Pellicer and Leitch, 2020).

**FIGURE 1:**
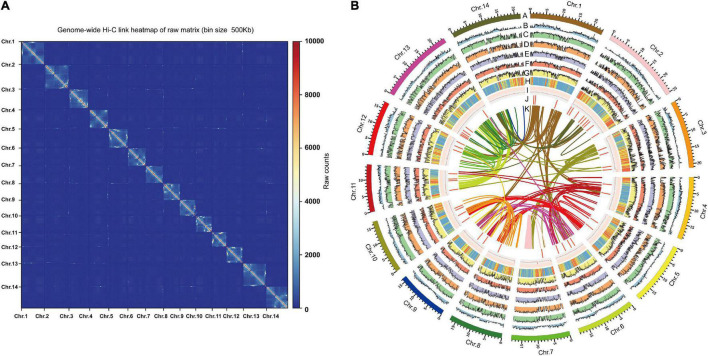
Genome features of genome assembly of *A. indica.* **(A)** Hi-C contact data mapped on the *A. indica* genome showing genome-wide all- by-all interactions. **(B)** The landscape of genome assembly and annotation of *A. indica*. (A) Circular representation of the pseudomolecules. (B) The distribution of gene density with densities calculated in 500 kb windows. (C–G) Expression of *A. indica* genes (from outside to inside tracks: stem, root, leaf, fruit and flower). (H,I) The distribution of repeat density and GC density with densities calculated in 500 kb windows. (J) Locations of genes mapped to secondary metabolism. (K) Syntenic blocks.

We further conducted the Hi-C sequencing to scaffold the preliminary assemblies and enhance the assembled contiguity at the chromosome level. In total, the Hi-C sequencing generated approximately 40.48 Gb clean reads. 94.5% reads from Hi-C sequencing were mapped to the assembled contigs, of which 26.1% were unique mapped read pairs (Supplementary Table 1). The verified read pairs were selected after considering the map position and orientation of the unique mapped read pairs. Then, according to the contiguity information between Hi-C read pairs, ALLHIC software was used to cluster, order, and orient the previous assemblies for chromosome-level scaffolding (Figure 1A). A total of 70 scaffolds were obtained after Hi-C sequencing reads assist chromosome assemble, of which 14 scaffolds formed chromosomes (Figure 1B and Supplementary Table 1). The final size of the *A. indica* genome assembly was 281 Mb, and the scaffold N50 was 19 Mb (Table 1).

**TABLE 1:** Statistics of the *A. indica* genome assembly.

Feature	Value
Genome size (Mb)	281
Genome GC%	32.2
N50 (Mb)	19
Gene number	25,767
Average gene length (bp)	2,837
Exon no. per gene	5.4
Exon number	138,941
Average exon length (bp)	231
Total exon length (bp)	32,191,037

### Evaluation of the Genome Assembly

The quality of the assembly was assessed and compared with the reference genome sequence of *A. indica* from NCBI (GCA_000439995.3) (Supplementary Figure 3 and Table 2). The Benchmarking Universal Single-Copy Orthologs (BUSCO) (Simao et al., 2015) analysis was used to evaluate the integrity of the genome. The BUSCO assessment showed that the completeness of the assembled genome of *A. indica* was 91.7%, which was much higher than that of the reference genome (Supplementary Figure 3A, Table 2, and Supplementary Table 2). The average LAI score of *A. indica* genome was 4.82, which was lower than the “reference” quality (10 < LAI < 20) based on the LAI classification (Ou et al., 2018). The Illumina short reads were also used to assess the integrity of the genome. The transcriptomic Illumina sequencing short reads were mapped to the two assemblies by Hisat2 (Kim et al., 2015), and approximately 92.76 and 87.49% of the reads were mapped to our assembly and GCA_000439995.3, respectively. By using Bowtie2 (Langmead and Salzberg, 2012) software, the genomic Illumina sequencing short reads were also mapped to the assemblies. About 99.29 and 97.09% of the Illumina short reads could map to our assembly and GCA_000439995.3, respectively (Supplementary Figure 3B). Finally, collinearity analysis revealed good collinearity between our assembly and GCA_000439995.3 (Supplementary Figure 3C).

**TABLE 2:** Comparison of the *A. indica* genome assembly versions.

Feature	This study	Krishnan et al., 2012	GCA_000439995.3
Sequence technology	Illumina + PacBio + Hi-C	Illumina + PacBio	Illumina
Assembly level	Chromosome	Scaffold	Contig
Genome size (Mb)	281	216	264
Genome GC%	32.2	31.9	32.0
Number of scaffolds	70	25,560	126,142
Scaffold N50 (bp)	19,542,739	2,629,187	3,491
Number of contigs	870	48,555	142,701
Contig N50 (bp)	6,039,544	25,406	3,310
BUSCO	91.7%	91.4%	79.9%

### Gene Prediction and Genome Annotation

Gene models were generated by a combination of reference plant protein homology support, transcriptome data, and *ab initio* gene prediction. All gene models were merged with MAKER (Holt and Yandell, 2011), resulting in a total of 25,767 protein-coding genes with an average sequence length of 2,837 bp. On average, each predicted gene contained 5.4 exons with a mean sequence length of 231 bp (Table 1). In addition, 3,856 non-coding RNAs, including 1,381 rRNAs, 1,204 tRNAs, 173 microRNAs (miRNAs), and 1,098 small nuclear RNAs (snRNAs) were identified (Supplementary Table 3). We also identified 40.99% of the assembled sequences as repetitive sequences, which was higher than that of the reported genomes (Kuravadi et al., 2015; Krishnan et al., 2016). The majority of the repeats were long terminal repeats (LTRs), constituting 16.88% of the genome. Unclassified elements, DNA elements, and long interspersed nuclear elements (LINEs), accounted for 14.28, 6.54, and 1.08% of the genome, respectively (Supplementary Table 4).

To further evaluate the functional validity of the predicted genes, Diamond, BLASTP, InterProScan and EggNOG-mapper were utilized by searching the Nr, SwissProt, InterPro, and EggNOG databases (Supplementary Figure 3D). Overall, 24,801 genes (96.2%) were functionally assigned. 95.4 and 81.6% of these genes found homologies and annotated proteins in the Nr and SwissProt databases, respectively. 84.3% of the genes were detected with conserved protein domains using InterProScan. In addition, 47.4% of the genes were categorized by Kyoto Encyclopedia of Genes and Genomes (KEGG) pathway (Moriya et al., 2007; Supplementary Table 5).

### Phylogenetic Analysis

To investigate the genetic diversity and evolutionary history of *A. indica* genome, a gene family clustering analysis with the *A. indica* genome and 13 other representative plant species was performed. These selected species included two plants in the *Sapindales* order (*Acer yangbiense* and *Citrus sinensis*), eight plants in the eudicot clade (*Arabidopsis thaliana*, *Theobroma cacao*, *Gossypium raimondii*, *Carica papaya*, *Vitis vinifera*, *Cucumis sativus*, *Fragaria vesca*, *Prunus persica*, and *Solanum lycopersicum*), and two outgroup species (*Brachypodium distachyon* and *Amborella trichopoda*).

**FIGURE 2:**
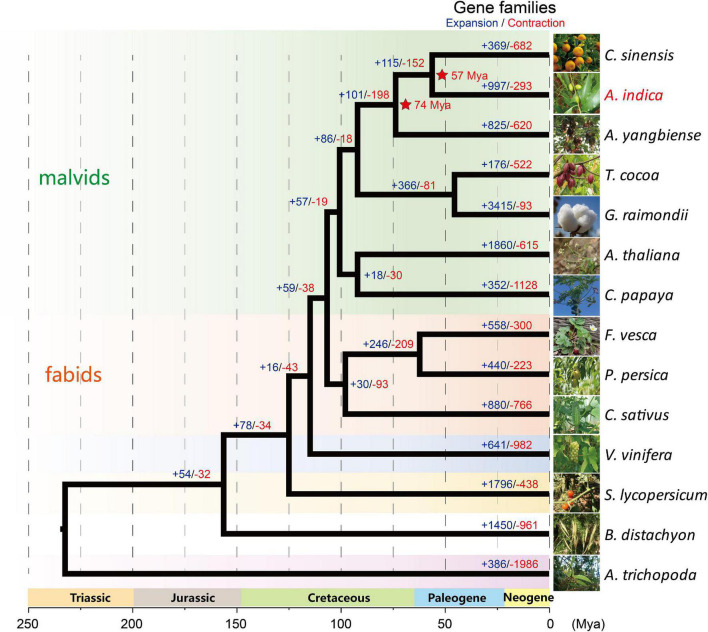
Maximum Likelihood based phylogenetic analysis of *A. indica* and 13 other plant species using 1,338 single-copy orthologous genes with PhyML software (Guindon et al., 2009). The estimation of the divergence times (Mya; red; star) of *A. indica* with its closely related species are indicated. The number of expanded gene families (+; blue) and the number of contracted gene families (–; red) are shown in each branch.

OrthoFinder (Emms and Kelly, 2019) was used to construct a phylogenetic tree with 1,338 single-copy orthologous genes among 14 species, which showed that *A. indica* is most closely related to *C. sinensis* (Figure 2). Further analysis showed that 36 gene families were specific to *A. indica* (Supplementary Table 6). Enrichment analysis showed that these specific genes were mostly involved in “binding,” “catalytic activity,” “metabolic process,” “cellular process,” and “membrane” (Supplementary Table 7). With the divergence time between *P. persica* and *F. vesca* as a calibration point (with the corrected time obtained from TimeTree Kumar et al. (2017)), the divergence time among these species were also estimated. *A. indica* and *C. sinensis* diverged from a common ancestor ∼57 Mya (Figure 2). To better understand the genetic basis of *A. indica*, the expansion and contraction of gene families were investigated. 997 gene families were expanded in *A. indica*, while 293 gene families were contracted from the *A. indica* genome. Compared with *C. sinensis*, which has 369 expanded gene families and 682 contracted gene families, *A. indica* has expanded more gene families. GO and KEGG analysis of the expanded and contracted gene families were also performed (Supplementary Figure 4 and Supplementary Table 8). The *A. indica*-specific expanded and contracted gene families might be related to the adaptation to *A. indica*-specific tropical niches. Further researches are required to verify the function of these genes.

### Genome Duplication Analysis

**FIGURE 3:**
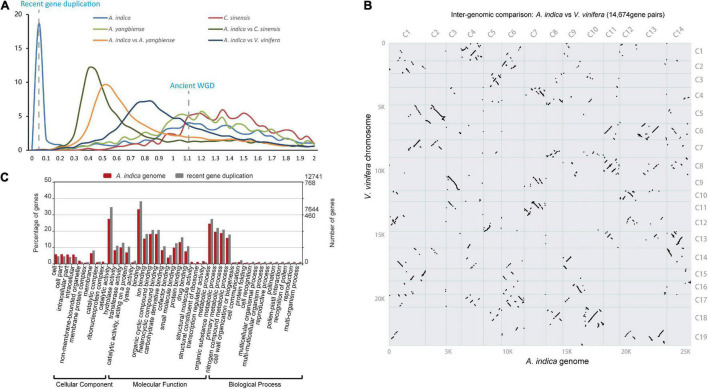
Genome evolution of *A. indica*. **(A)** *Ks* distribution (The gene pairs located in syntenic blocks) between syntenic genes within the *A. indica* genome or between genomes. **(B)** Inter-genomic syntenic analysis between *A. indica* genome and *V. vinifera* genome. **(C)** GO enrichment of the *A. indica* genome (green) and genes retained after recent gene duplication (orange) (*P* < 0.05). *Pearson chi-square test *P*-value < 0.05; **Pearson chi-square test *P*-value < 0.01.

To investigate genome wide duplications in *A. indica* genome, self-comparison of the *A. indica* genome was performed using MCScan (Tang et al., 2008; Supplementary Figure 5). 242 homologous blocks were identified in the intragenomic gene synteny of *A. indica*, containing 2,281 gene pairs. These homologous blocks were distributed across the 14 chromosomes, covering 17.66% of protein-coding genes (4,139/25,767). The synonymous nucleotide substitutions (*Ks*) of the gene pairs peaked at approximately 0.01 and 1.12 (Figure 3A). The first peak at approximately 1.12 indicated the core eudicot γ triplication event (∼165 Mya). The second peak at approximately 0.01 indicated a relatively recent duplication event or events. To distinguish whether this peak represents a whole genome duplication event or background duplications, we performed synteny analysis on *A. indica*, *V. vinifera*, *C. sinensis*, and *A. yangbiense* genomes (Figure 3B and Supplementary Figure 6). Intergenomic collinearity analysis showed 611 homologous blocks containing 14,674 gene pairs and a 3:3 syntenic relationship between *A. indica* and *V. vinifera* (Figure 3B and Supplementary Figure 7A). Although there were 2:1 syntenic relationship between *A. indica* vs. *C. sinensis* and *A. indica* vs. *A. yangbiense* (Supplementary Figures 7B,C), only 13 and 14% of the *A. indica* gene models in syntenic blocks, respectively, were present as two copies. Meanwhile, we did not identify large *C. sinensis* and *A. yangbiense* segments that have two syntenic copies in *A. indica* by the synteny dot plot of *A. indica* vs. *C. sinensis* and *A. indica* vs. *A. yangbiense* (Supplementary Figure 6). Our analysis indicated that *A. indica* didn’t experience additional WGD after the γ event, but a recent small-scale segmental duplication (Xu et al., 2013; Yang et al., 2019). The calculation of *Ks* for *A. indica* vs. *C. sinensis* indicated that this recent segmental duplication event occurred approximately 1.5 Mya. Furthermore, we also performed paralog analysis in *A. indica* genome using reciprocal best hits (RBH) from primary protein sequences by all-vs.-all BLASTp matches. We detected 6,298 RBH paralogous gene pairs in the *A. indica* genome, and the RBH paralog *Ks* distribution shows a *Ks* peak at around 0.01 (Supplementary Figure 8). That this RBH *Ks* peak is close to the syntelog *Ks* peak also indicates *A. indica* has a recent segmental duplication mixed with gene duplication.

Generally, gene duplication events vary the genomic architecture, including genome size, genome density, gene content, and gene expression. In this study, we defined the RBH paralogous gene pairs with the median *Ks* < 0.05 as the retained genes from recent gene duplication. A total of 768 gene pairs were retained after recent gene duplication. GO analysis revealed that these gene pairs were significantly involved in binding, catalytic activity, metabolic process, cellular process, and reproductive process (Figure 3C). Recent gene duplication may also affect the percentage of genes in many function categories with different contributions. In the *A. indica* genome, the percentage of retained genes from recent gene duplication in “catalytic activity GO:0003824,” “recognition of pollen GO:0048544,” “pollen-pistil interaction GO:0009875,” “pollination GO:0009856,” and “multi-multicellular organism process GO:0044706” was greater than that of the average genome content (Figure 3C and Supplementary Figure 9). We further calculated the omega values (*Ka*/*Ks*) for most of the homologous gene pairs. Most of the omega values for the homologous gene pairs were smaller than 1, which indicated that purifying selection may be the predominant action within the retained genes from recent gene duplication (Stix, 1992). However, 120 gene pairs were identified that have experienced potential positive selection. GO analysis showed that these genes were mainly enriched in “catalytic activity, acting on a protein GO:0140096,” “protein binding GO:0005515,” “protein-containing complex GO:0032991,” and “membrane-bounded organelle GO:0043227” (Supplementary Table 9 and Supplementary Figure 10).

### Secondary Metabolite Analysis

**FIGURE 4:**
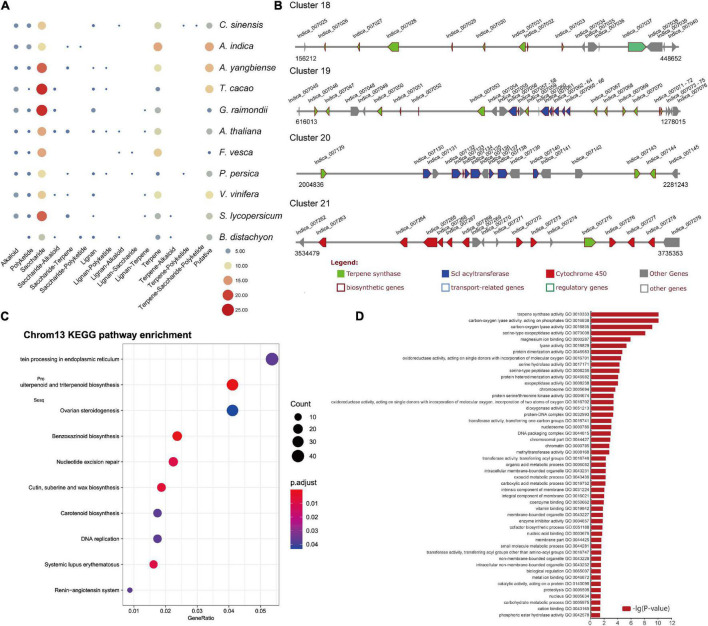
Secondary metabolite analysis. **(A)** Secondary metabolite analysis of *A. indica* and other 11 species. **(B)** The organization and architecture of terpene-related gene clusters on chromosome 13. **(C)** Dot plot of the KEGG pathway enrichment analysis of genes on chromosome 13. **(D)** The GO enrichment analysis of genes on chromosome 13 (*P* < 0.05).

Genes encoding some specialized metabolic pathways are found physically clustered in plant genomes (Nutzmann et al., 2016; Liu et al., 2020). We utilized the PlantiSMASH analytical pipeline (Hu et al., 2019) to identify physically clustered specialized metabolic pathway genes. According to the analysis, 50 clusters including 692 genes were identified in the *A. indica* genome (Supplementary Table 10). The sizes of the identified clusters range from 27.2 to 1634.4 kb. 105 (out of 692) clustered genes were contained in the 997 *A. indica*-specific expansion gene families (Supplementary Table 10). Furthermore, 41 (*C. sinensis*), 51 (*A. yangbiense*), 48 (*T. cocoa*), 47 (*G. raimondii*), 45 (*A. thaliana*), 35 (*F. vesca*), 33 (*P. persica*), 30 (*C. sativus*), 46 (*V. vinifera*), 47 (*S. lycopersicum*), and 29 (*B. distachyon*) clusters were detected in other 11 species (Figure 4A). As expected, more terpene-related clusters were identified in the *A. indica* genome than that of other species.

Azadirachtin is a triterpenoid compound of neem tree, which has effective insecticidal activities against a wide range of insect species, but has very low toxicity to mammals. Terpene synthase (TPS), cytochrome P450 (CYP450), alcohol dehydrogenase (ADH), acyltransferase (ACT), and esterase (EST) were proposed to be involved in biosynthesis of azadirachtin (Wang et al., 2020). In this study, a large number of genes encoding CYP 450s (78), TPSs (58), and ACTs (34) were identified in secondary metabolite biosynthesis gene clusters. Genes encoding ADHs, and ESTs may reside dispersed in the genome. The terpene-related clusters mainly distributed on chromosome 1, 2, 3, 5, 6, 7, 10, 11, 12, and 13. Four terpene-related clusters (cluster 18–21) covering ∼1.4 Mb were distributed on chromosome 13 (Figure 4B). Among the 83 clustered terpene-related genes on chromosome 13, 12 genes were contained in the *A. indica*-specific expanded gene families. These genes are proposed to be potential genes participated in the terpene biosynthesis specific to *A. indica*.

KEGG enrichment analysis was performed to investigate the function of genes on chromosome 13. The result showed that genes on chromosome 13 were mainly involved in “Protein processing in endoplasmic reticulum,” “Sesquiterpenoid and triterpenoid biosynthesis,” and “Ovarian steroidogenesis” (Figure 4C). Furthermore, when we performed GO enrichment analysis using the genes on chromosome 13, the genes associated with “terpene synthase activity” (GO:0010333) exhibited a low *P*-value, indicating that “terpene synthase activity” was the most enriched functional category of chromosome 13 (Figure 4D).

### Terpene Synthase Gene Family

**FIGURE 5:**
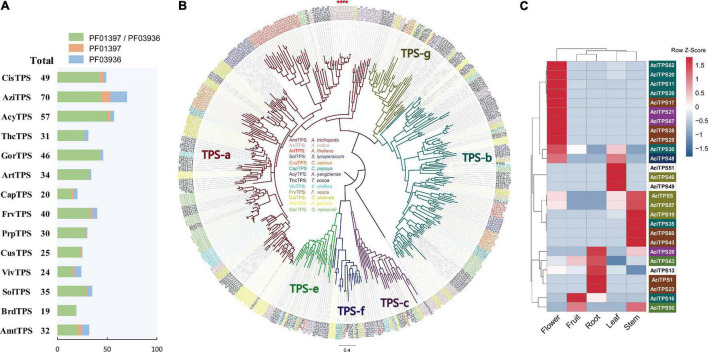
Analysis of TPS gene family in *A. indica*. **(A)** Statistics of predicted TPS genes in *A. indica* and other 13 plants. **(B)** Phylogenetic analysis of TPS family members based on the protein sequences. A Maximum-Likelihood (ML) tree was generated from an alignment of 409 TPS protein sequences, comprising 403 putative TPSs from *A. indica* and other 13 plants (the remaining TPS genes were too short for meaningful alignment) and other six reported TPS genes belonged to TPS- a (AAX16121.1), b (AAQ16588.1), c (AAD04292.1), e (Q39548.1), f (Q93YV0.1), and g (ADD81294.1) subfamilies. The *A. indica* TPS genes are highlighted by red stars. **(C)** *A. indica* TPS genes substantially expressed in five tested organs (root, flower, fruit, leaf, and stem). The color of TPS genes depends on the subfamily information.

TPS gene family is characterized by two large domains: PF01397 (Terpene synthase, N-terminal domain) and PF03936 (Terpene synthase family, metal binding domain). To investigate the characteristics and evolution of the TPS gene families, we identified a total of 512 putative TPS genes in *A. indica* and other 13 plant genome. 70 putative TPS genes were identified in *A. indica*; These consisted of 44 AziTPS genes containing both PF01397 and PF03936 domains, nine AziTPS genes containing PF01397 domain, and 17 AziTPS genes containing PF03936 domain. *A. indica* (*N* = 70) contained the most copies of TPSs compared with other plants, followed by *C. sinensis* (*N* = 49) and *A. yangbiense* (*N* = 57) (Figure 5A and Supplementary Table 11). In addition, eight AziTPS genes experienced recent gene duplication (Supplementary Table 11).

Phylogenetic analysis was performed using 403 TPSs (the remaining TPS genes were too short for meaningful alignment) from *A. indica* and other 13 plants, including six reported TPS genes belonged to TPS- a, b, c, e, f, and g subfamilies, respectively (Supplementary Table 11). As shown in Figure 5B, the topology of six subfamilies is similar to that of the previous papers (Kumar et al., 2018b; Zhou et al., 2020; Ji et al., 2021). Among the 40 AziTPS used in phylogenetic analysis, 15, 13, 4, 3, 1, and 4 AziTPS genes fell in TPS- a, b, c, e, f, and g subfamilies, respectively. TPS-a and -b subfamilies were the main subfamilies in *A. indica*, approximately 37.5 and 32.5% of the total AziTPS genes in phylogenetic analysis. This is in accordance with other plant species, including tea, grape, and Chinese mahogany (Chen et al., 2011; Zhou et al., 2020; Ji et al., 2021). Furthermore, we identified putative *A. indica*-specific TPSs using phylogenetic analysis and a cutoff of 55% identity, which indicates separate subfamily assignment (Liu et al., 2018; Tu et al., 2020). A total of nine *A. indica*-specific TPS genes were identified (Supplementary Table 11). Interestingly, seven of these specific AziTPSs (Indica_007028, Indica_007047, Indica_007053, Indica_007068, Indica_007070, Indica_007072, and Indica_007143) were located in the terpene-related clusters (cluster 18, 19, and 20) of chromosome 13 (Supplementary Table 11).

We further investigated the expression pattern of TPS genes in *A. indica*. Transcriptome datasets from five tissues of *A. indica* were obtained from our previous study (Wang et al., 2020) and remapped to the chromosome-level genome assembly in this study. More than 88% of the RNAseq reads were mapped uniquely to the genome assembly across all samples (Supplementary Table 12). Transcripts of 27 TPS genes were detected in the tested tissues. Most of the detected transcripts exhibited a spatial-specific expression pattern (Figure 5C). Nine, one, four, three, and four genes were exclusively expressed in flower, fruit, root, leaf, and stem, respectively. Seven genes (AziTPS30, −48, −5, −57, −26, −63, and −50) were primarily expressed in one or two tissues.

### Cytochrome P450 Gene Family

**FIGURE 6:**
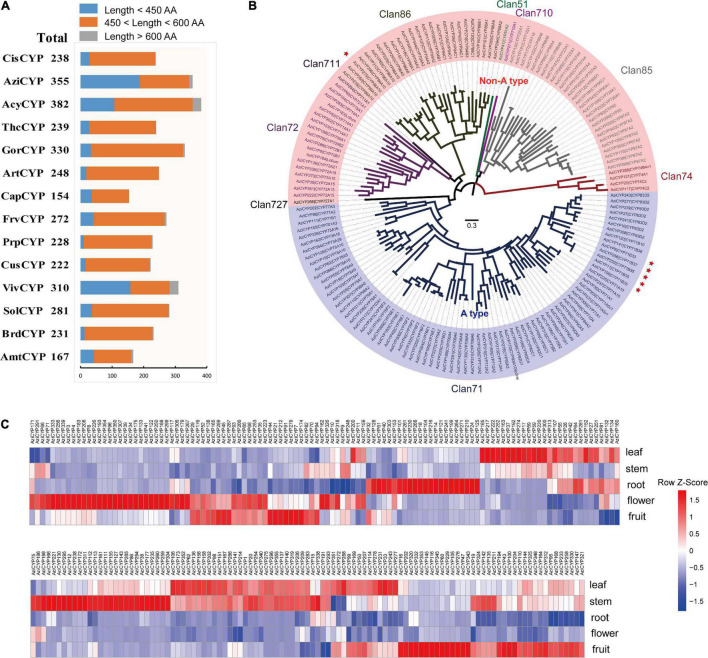
Analysis of cytochrome P450 gene family in *A. indica*. **(A)** Statistics of predicted cytochrome P450 genes in *A. indica* and other 13 plants. **(B)** Phylogenetic analysis cytochrome P450 family members in *A. indica* based on the protein sequences. A ML tree was generated from an alignment of 156 *A. indica* cytochrome P450 protein sequences (450 < length < 600). The entire family of cytochrome P450 genes is shown for each clan next to the tree with different color. The *A. indica* P450 genes are highlighted by red stars. **(C)** *A. indica* CYP genes substantially expressed in five tested organs (fruit, flower, root, stem, and leaf).

The characteristics and evolution of the cytochrome P450 (CYP) gene families were also investigated. In total, 3,657 CYP genes were identified from all 14 plant genomes (Figure 6A and Supplementary Table 13). A total of 355 CYP genes were in the *A. indica* genome, of which 36 CYP genes were involved in recent gene duplication (Supplementary Table 13). Moreover, 157 full length CYP (450 < length < 600) protein sequences of *A. indica* were aligned to construct a phylogenetic tree. As shown in Figure 6B, the phylogenetic tree was divided into two major clades: A type (49%; 77/157) and non-A type (51%; 80/157); and further clustered into nine clans. The Clan 71 is the largest clan and comprises of 49% (77/157) members; 18, 4, 28, and 25 members are classified into Clan72, Clan74, Clan85, and Clan86; remaining Clan51, Clan710, Clan711, and Clan727 are single family clans.

In order to identify putative *A. indica*-specific CYP genes, we constructed a phylogenetic tree using amino acid sequence alignment of 2,807 (450 < length < 600) CYP genes in *A. indica* and other 13 plants genome with a cutoff of 55% identity (Liu et al., 2018; Tu et al., 2020). Six *A. indica-*specific CYP genes were identified (Supplementary Table 13). Similar to TPS genes, five of these CYP genes (Indica_007272, Indica_007273, Indica_007276, Indica_007277, and Indica_007278) were located in the terpene-related cluster 21 of chromosome 13 (Supplementary Table 13). These specific-TPSs and CYPs in the terpene-related secondary metabolite biosynthesis gene clusters of chromosome 13 might be involved in the specific biosynthesis of azadirachtin.

We also investigated the expression pattern of *A. indica* CYP genes in different tissues (fruit, flower, root, stem, and leaf). Transcripts of 221 CYP genes were detected with different patterns (Figure 6C). There were more high-expressed CYPs in fruit, stem and leaf than flower and root. The high-expressed CYPs in fruit, stem and leaf were 83, 88, and 97, respectively. CYPs with a high-expression in the tissues (fruit and leave) with high azadirachtin. A content, are more likely to be involved in azadirachtin biosynthesis. Furthermore, *A. indica*-specific AziCYP256 (Indica_007272) and AziCYP8 (Indica_007273) were highly expressed in fruit and flower.

### Resistance Genes

Plants have developed a wide range of defense mechanisms to protect themselves against the attack of pathogens in their constant struggle for survival. In general, proteins encoded by resistance (R) genes display modular domain structures. In this study, putative R genes in the *A. indica* genome (1,488) and other 13 species were identified (Supplementary Table 14). In the *A. indica* genome, 238 R genes may exert their disease resistance function as cytoplasmic protein through canonical resistance domains, such as the nucleotide-binding sites (NBSs), the leucine-rich repeat (LRR), and terminal inverted repeat (TIR) domains (Supplementary Table 14). 167 NBS genes were identified in the *A. indica* genome, which could be divided into five classes according to the conserved domains: N, CN, CNL, NL, and TNL. The majority were N type which contained only the NB-ARC domain. In comparison with other genomes in malvids, most of the NBS genes in the *A. indica* genome were underrepresented relative to other Sapindales genomes (*C. sinensis* and *A. yangbiense*) and Malvales genomes (*T. cacao* and *G. raimondii*), but overrepresented relative to other Brassicales genome (*A. thaliana* and *C. papaya*). In addition, 447 genes were classified as transmembrane receptors, including 221 receptor-like kinases (RLK), and 226 receptor-like proteins (RLP). 721 putative kinases were also identified in the *A. indica* genome.

## Discussion

*A. indica* is a valuable plant species given its economic and pharmaceutical significance (Stix, 1992). A high-quality reference genome is essential for the genetic and genomic studies of *A. indica*. However, molecular-level studies on this species are limited. Here, we assembled the first chromosome-scale genome of *A. indica* by a combination of Illumina, PacBio, and Hi-C technology. The size of the genome assembly is approximately 281 Mb, with a scaffold N50 value of 19 Mb. The N50 of our assembled genome is much higher than that of the previous published draft genomes (Krishnan et al., 2012, 2016; Kuravadi et al., 2015). Our assembled genome size covered ∼73.2% of the estimated genome size (385 Mb) by flow cytometry. However, previously assembled 12 contig-level *A. indica* genomes were generally less than 300 Mb (Krishnan et al., 2016). The *A. indica* genome shows a high level of heterozygosity (0.896%) and repeat content (40.99%), rendering substantial challenges for its assembly (Nowak et al., 2015). Hi-C technology has been broadly available for many complex species (Chen et al., 2020). In this study, Hi-C technology facilitated the completeness and accuracy of a chromosome-level genome assembly for *A. indica*. The improvement of BUSCO evaluation shows that our assembly represents a better template for gene annotation than the reference sequence. Considering that the genome is highly heterozygous and repetitive, the present version represents a high-quality genome assembly. The obtained genome is also the second chromosome-level genome of the Meliaceae family, which will pave the way for further genetic and genomic studies of this family.

Gene duplication is an important evolutionary force that provides abundant raw materials for genetic novelty, morphological diversity and speciation (Qiao et al., 2018). In this study, we find no evidence that *A. indica* experienced WGD after the ancient γ event shared by all eudicots. However, recent gene duplication events mixed with small-scale segmental duplication likely affected multiple genes in *A. indica*. This may also explain the fact that *A. indica* had more expanded gene families than *C. sinensis*. Our result is in agreement with the research of Chinese mahogany, which indicated that a recent WGD occurred in *Toona sinensis* (Ji et al., 2021). The occurrence of recent WGD mixed with gene duplications has been reported in *Papaver somniferum* L. genome (Guo et al., 2018). Furthermore, recent WGD was also observed in *Panax notoginseng* genome (Jiang et al., 2021). All these results are highly benefit for in-depth investigation of the survival and diversification history the of Meliaceae family.

Limonoids are natural triterpenoid products made by plants of the Meliaceae family. They are known for their insecticidal activity and potential pharmaceutical properties. *A. indica* is known as the reservoir of azadirachtin, the most famous limonoid insecticide. Secondary metabolite analysis revealed that *A. indica* contained more terpene-related clusters than that of the other 11 species. Eighty three (out of 247) clustered terpene-related genes were located on chromosome 13. The KEGG pathway enrichment analysis revealed that 33 genes were correlated with the “Sesquiterpenoid and triterpenoid biosynthesis” pathway. These results indicated that chromosome 13 may have played a central role in the evolution of terpenoid biosynthetic machinery in *A. indica*.

The TPS and CYP gene families are responsible for the biosynthesis of terpenoids in plants. 70 TPS genes were identified in *A. indica*, which is much more than that of the other 13 species. This is consistent with the result of Chinese mahogany (*T. sinensis*), the first chromosome-level genome assembly of the Meliaceae family (Ji et al., 2021). Furthermore, TPS genes have also been reported to be abundant in other angiosperms that are rich in terpenoids. For example, the *Nymphaea colorata* genome harbored 92 putative TPS genes, mainly consisting of copies from subfamily TPS-b, with no TPS-a copies (Zhang et al., 2020). In contrast, more than a dozen TPS-a genes were identified in the *A. indica* genome. These TPS-a genes might be responsible for sesquiterpene biosynthesis in *A. indica*. In addition, 355 CYP genes were identified in *A. indica*, six of which were *A. indica* specific CYPs. The expansion of terpene-related gene clusters, TPSs and CYPs, may promote the formation of terpenoids in *A. indica*. A total of eight TPS genes and 36 CYP genes were involved in recent gene duplication, suggesting that recent gene duplication event may have been responsible for terpenoid biosynthesis-related gene expansion in *A. indica*, after its split from *C. sinensis*. Notably, most of the identified *A. indica* -specific TPSs and CYPs were located in the terpene-related clusters on chromosome 13, indicating that these regions were likely to be involved in azadirachtin biosynthesis. This study provided the first chromosome-level genome of *A. indica*, and a genomic perspective for the synthesis and evolution of azadirachtin.

## Data Availability Statement

Raw data from this study were deposited in the NCBI SRA (Sequence Read Archive) database under the Bioproject ID: PRJNA645650. The genome sequence data (Illumina, PacBio, and Hi-C data) are available under accession numbers SRR12315383, SRR12321691, and SRR12321285. The assembled genome was submitted to DDBJ/ENA/GenBank with accession number JAGQDM000000000.

## Author Contributions

YD and WS designed the project and wrote the draft manuscript. WS participated in the genome assembly and annotation. YD, SW, JL, and ZY contributed to the genome evolution analysis, gene family analysis, and resistance gene identification. NW, HJ, JQ, and Y-XH revised the manuscript. All authors read and approved the final manuscript.

## Conflict of Interest

The authors declare that the research was conducted in the absence of any commercial or financial relationships that could be construed as a potential conflict of interest.

## Publisher’s Note

All claims expressed in this article are solely those of the authors and do not necessarily represent those of their affiliated organizations, or those of the publisher, the editors and the reviewers. Any product that may be evaluated in this article, or claim that may be made by its manufacturer, is not guaranteed or endorsed by the publisher.

## Footnotes

1
http://cegg.unige.ch/orthodb

2
https://github.com/galaxyproject/tools-iuc/tree/master/tools/minimap2

3
https://github.com/tpoorten/dotPlotly

4
https://www.girinst.org/repbase/

5
https://www.ncbi.nlm.nih.gov/protein/

6
https://www.ebi.ac.uk/interpro/

7
http://www.uniprot.org
