# Supplementary material for: Genomic Analysis Based on Chromosome-Level Genome Assembly Reveals an Expansion of Terpene Biosynthesis of Azadirachta indica
Source: Front Plant Sci. 2022 Apr 18;13:853861. doi: 10.3389/fpls.2022.853861 (PMC9069239; doi:10.3389/fpls.2022.853861)
Supplement: Supplementary file 3 [file Table_2.docx]

**Supplementary Table 2**. Quality assessment of the assembled genome of *A. indica* using BUSCOs.

| **Type** | **Number** | **Percent (%)** |
| --- | --- | --- |
| Complete BUSCOs (C) | 278 | 91.7 |
| Complete and single-copy BUSCOs (S) | 250 | 82.5 |
| Complete and duplicated BUSCOs (D) | 28 | 9.2 |
| Fragmented BUSCOs (F) | 2 | 0.7 |
| Missing BUSCOs (M) | 23 | 7.6 |
| Total BUSCO groups searched | 303 | 100 |
