# Supplementary material for: Genomic Analysis Based on Chromosome-Level Genome Assembly Reveals an Expansion of Terpene Biosynthesis of Azadirachta indica
Source: Front Plant Sci. 2022 Apr 18;13:853861. doi: 10.3389/fpls.2022.853861 (PMC9069239; doi:10.3389/fpls.2022.853861)
Supplement: Supplementary file 4 [file Table_3.docx]

**Supplementary Table 3**. Statistics on the annotation of non-coding RNA of the *A. indica* genome.

| **Type** |  | **Copy** | **Total length (bp)** | **% of genome** |
| --- | --- | --- | --- | --- |
| miRNA |  | 173 | 21342 | 0.007596 |
| tRNA |  | 1204 | 90808 | 0.032321 |
| rRNA |  | 1381 | 1895631 | 0.674716 |
| snRNA | CD-box | 408 | 41730 | 0.014853 |
|  | HACA-box | 44 | 5653 | 0.002012 |
|  | splicing | 97 | 14494 | 0.005158 |
