# Supplementary material for: Genomic Analysis Based on Chromosome-Level Genome Assembly Reveals an Expansion of Terpene Biosynthesis of Azadirachta indica
Source: Front Plant Sci. 2022 Apr 18;13:853861. doi: 10.3389/fpls.2022.853861 (PMC9069239; doi:10.3389/fpls.2022.853861)
Supplement: Supplementary file 5 [file Table_4.docx]

**Supplementary Table 4**. Repeats in the *A. indica* genome assembly.

| **Types** | **No. of copies** | **Length (bp)** | **Coverage of genome (%)** |
| --- | --- | --- | --- |
| DNA transposon | 58968 | 18378069 | 6.54 |
| LINE | 8035 | 3034159 | 1.08 |
| Long terminal repeat | 66790 | 47418507 | 16.88 |
| SINE | 5 | 394 | 0.00 |
| Other∗ | 2925 | 6226012 | 2.21 |
| Unknown | 130734 | 40113882 | 14.28 |
| Total | 267457 | 115171023 | 40.99 |

∗Other includes microsatellites, simple repeats, and low-complexity sequences.
