# Supplementary material for: Genomic Analysis Based on Chromosome-Level Genome Assembly Reveals an Expansion of Terpene Biosynthesis of Azadirachta indica
Source: Front Plant Sci. 2022 Apr 18;13:853861. doi: 10.3389/fpls.2022.853861 (PMC9069239; doi:10.3389/fpls.2022.853861)
Supplement: Supplementary file 6 [file Table_5.docx]

**Supplementary Table 5**. Summary of the functional annotation in *A. indica* genome.

| **Annotation database** | **Annotated number** | **Percentage (%)** |
| --- | --- | --- |
| Nr | 24,589 | 95.4% |
| SwissProt | 21,024 | 81.6% |
| EggNOG | 24,448 | 94.8% |
| COG | 22,789 | 88.4% |
| InterPro | 21,740 | 84.3% |
| GO | 16,170 | 62.7% |
| KEGG Pathway | 12,126 | 47.4% |
| Total | 24,801 | 96.2% |
