# Supplementary material for: Genomic Analysis Based on Chromosome-Level Genome Assembly Reveals an Expansion of Terpene Biosynthesis of Azadirachta indica
Source: Front Plant Sci. 2022 Apr 18;13:853861. doi: 10.3389/fpls.2022.853861 (PMC9069239; doi:10.3389/fpls.2022.853861)
Supplement: Supplementary file 7 [file Table_6.docx]

**Supplementary Table 6**. Summary of gene family clustering in *A. indica*.

| **Species** | **Total**  **genes** | **Genes in families** | **Family** | **Unclustered genes** | **Species-specific families** | **Genes per family** |
| --- | --- | --- | --- | --- | --- | --- |
| *A. indica* | 25657 | 22384 | 13398 | 3273 | 36 | 1.67 |
| *A. yangbiense* | 27760 | 24057 | 12931 | 3703 | 187 | 1.86 |
| *C. sinensis* | 20286 | 19367 | 12000 | 919 | 23 | 1.61 |
| *A. thaliana* | 27444 | 22584 | 12286 | 4860 | 347 | 1.84 |
| *T. cacao* | 21257 | 20734 | 13324 | 523 | 17 | 1.56 |
| *G. raimondii* | 35177 | 31903 | 13446 | 3274 | 399 | 2.37 |
| *C. papaya* | 18003 | 16840 | 12404 | 1163 | 19 | 1.35 |
| *V. vinifera* | 23647 | 18177 | 11952 | 5470 | 72 | 1.52 |
| *C. sativus* | 19521 | 18580 | 12174 | 941 | 62 | 1.53 |
| *F. vesca* | 24056 | 22166 | 13083 | 1890 | 91 | 1.69 |
| *P. persica* | 22988 | 21826 | 13186 | 1162 | 58 | 1.66 |
| *S. lycopersicum* | 25157 | 23040 | 12532 | 2117 | 210 | 1.84 |
| *B. distachyon* | 25447 | 20039 | 11586 | 5408 | 670 | 1.73 |
| *A. trichopoda* | 17099 | 15775 | 12042 | 1324 | 178 | 1.31 |
