# Supplementary material for: Genomic Analysis Based on Chromosome-Level Genome Assembly Reveals an Expansion of Terpene Biosynthesis of Azadirachta indica
Source: Front Plant Sci. 2022 Apr 18;13:853861. doi: 10.3389/fpls.2022.853861 (PMC9069239; doi:10.3389/fpls.2022.853861)
Supplement: Supplementary file 8 [file Table_7.docx]

**Supplementary Table 7**. The list of *A. indica* specific genes.

|  | **GO annotation** | | | **KEGG annotation** | |
| --- | --- | --- | --- | --- | --- |
| **Protein ID** | **Cellular Component** | **Molecular Function** | **Biological Process** | **KO number** | **Definition** |
| Indica_007398-RA |  | GO:0005524\|ATP binding; GO:0004672\|protein kinase activity; | GO:0006468\|protein phosphorylation; | K08829 | MAK; male germ cell-associated kinase [EC:2.7.11.22] |
| Indica_007413-RA |  | GO:0005524\|ATP binding; GO:0004672\|protein kinase activity; | GO:0006468\|protein phosphorylation; | K08829 | MAK; male germ cell-associated kinase [EC:2.7.11.22] |
| Indica_007414-RA |  | GO:0005524\|ATP binding; GO:0004672\|protein kinase activity; | GO:0006468\|protein phosphorylation; | K08829 | MAK; male germ cell-associated kinase [EC:2.7.11.22] |
| Indica_007416-RA |  | GO:0005524\|ATP binding; GO:0004672\|protein kinase activity; | GO:0006468\|protein phosphorylation; | K08829 | MAK; male germ cell-associated kinase [EC:2.7.11.22] |
| Indica_007418-RA |  | GO:0005524\|ATP binding; GO:0004672\|protein kinase activity; | GO:0006468\|protein phosphorylation; | K08829 | MAK; male germ cell-associated kinase [EC:2.7.11.22] |
| Indica_001271-RA |  | GO:0003824\|catalytic activity; |  |  |  |
| Indica_001274-RA |  | GO:0003824\|catalytic activity; |  |  |  |
| Indica_001279-RA |  | GO:0003824\|catalytic activity; |  |  |  |
| Indica_001282-RA |  | GO:0003824\|catalytic activity; |  |  |  |
| Indica_026282-RA |  |  |  |  |  |
| Indica_026294-RA | GO:0016020\|membrane; | | GO:0055114\|oxidation-reduction process; |  |  |
| Indica_026300-RA | GO:0016020\|membrane; | | GO:0055114\|oxidation-reduction process; |  |  |
| Indica_026387-RA | GO:0016020\|membrane; | | GO:0055114\|oxidation-reduction process; |  |  |
| Indica_013831-RA |  | GO:0016787\|hydrolase activity; |  |  |  |
| Indica_013832-RA |  | GO:0016787\|hydrolase activity; |  |  |  |
| Indica_013838-RA |  | GO:0016787\|hydrolase activity; |  |  |  |
| Indica_023874-RA |  | GO:0005515\|protein binding; |  |  |  |
| Indica_023876-RA |  | GO:0005515\|protein binding; |  |  |  |
| Indica_024678-RA |  | GO:0005515\|protein binding; |  |  |  |
| Indica_026283-RA |  |  |  |  |  |
| Indica_026289-RA |  |  |  |  |  |
| Indica_026299-RA |  |  |  |  |  |
| Indica_000067-RA |  | GO:0003676\|nucleic acid binding; |  |  |  |
| Indica_001559-RA |  | GO:0003676\|nucleic acid binding; |  |  |  |
| Indica_021414-RA |  | GO:0005524\|ATP binding; GO:0004672\|protein kinase activity; | GO:0006468\|protein phosphorylation; |  |  |
| Indica_021590-RA |  | GO:0005524\|ATP binding; GO:0004672\|protein kinase activity; | GO:0006468\|protein phosphorylation; |  |  |
| Indica_025821-RA |  |  |  |  |  |
| Indica_025823-RA |  | GO:0008194\|UDP-glycosyltransferase activity; | |  |  |
| Indica_011978-RA |  |  |  | K13343 | PEX14; peroxin-14 |
| Indica_026073-RA |  |  |  | K13343 | PEX14; peroxin-14 |
| Indica_009679-RA |  | GO:0043531\|ADP binding; |  |  |  |
| Indica_010481-RA |  | GO:0043531\|ADP binding; |  |  |  |
| Indica_018790-RA |  |  |  |  |  |
| Indica_019629-RA |  |  |  |  |  |
| Indica_016399-RA |  | GO:0005515\|protein binding; |  |  |  |
| Indica_017175-RA |  | GO:0005515\|protein binding; |  |  |  |
